# Health system's response for physician workforce shortages and the upcoming crisis in Ethiopia: a grounded theory research

**DOI:** 10.1186/s12960-017-0257-5

**Published:** 2017-12-28

**Authors:** Tsion Assefa, Damen Haile Mariam, Wubegzier Mekonnen, Miliard Derbew

**Affiliations:** 1 School of Public Health, Addis Ababa University, PO Box 9086, Addis Ababa, Ethiopia; 2 School of Medicine, Addis Ababa University, PO Box 9086, Addis Ababa, Ethiopia

**Keywords:** Flooding strategy, Grounded theory, Medical education expansion, System response/continuity

## Abstract

**Background:**

A rapid transition from severe physician workforce shortage to massive production to ensure the physician workforce demand puts the Ethiopian health care system in a variety of challenges. Therefore, this study discovered how the health system response for physician workforce shortage using the so-called flooding strategy was viewed by different stakeholders.

**Methods:**

The study adopted the grounded theory research approach to explore the causes, contexts, and consequences (at the present, in the short and long term) of massive medical student admission to the medical schools on patient care, medical education workforce, and medical students. Forty-three purposively selected individuals were involved in a semi-structured interview from different settings: academics, government health care system, and non-governmental organizations (NGOs). Data coding, classification, and categorization were assisted using ATLAs.ti qualitative data analysis scientific software.

**Results:**

In relation to the health system response, eight main categories were emerged: (1) reasons for rapid medical education expansion; (2) preparation for medical education expansion; (3) the consequences of rapid medical education expansion; (4) massive production/flooding as human resources for health (HRH) development strategy; (5) cooperation on HRH development; (6) HRH strategies and planning; (7) capacity of system for HRH development; and (8) institutional continuity for HRH development.

The demand for physician workforce and gaining political acceptance were cited as main reasons which motivated the government to scale up the medical education rapidly. However, the rapid expansion was beyond the capacity of medical schools’ human resources, patient flow, and size of teaching hospitals. As a result, there were potential adverse consequences in clinical service delivery, and teaching learning process at the present: “the number should consider the available resources such as number of classrooms, patient flows, medical teachers, library…”*.* In the future, it was anticipated to end in surplus in physician workforce, unemployment, inefficiency, and pressure on the system: “…flooding may seem a good strategy superficially but it is a dangerous strategy. It may put the country into crisis, even if good physicians are being produced; they may not get a place where to go…”*.*

**Conclusion:**

Massive physician workforce production which is not closely aligned with the training capacity of the medical schools and the absorption of graduates in to the health system will end up in unanticipated adverse consequences.

## Background

In Ethiopia, since the modern health service delivery has been introduced at the beginning of twentieth century to the present, there has been shortage of human resources for health. Besides, high physician out migration over the last several years coupled with longstanding low production of medical doctors hasten the demand for physician workforce [1, 2]. More recently, however, succeeding the “flooding strategy” recommendation by WHO and Global Health Workforce Alliance (GHWA) in 2010 [3] and in response to the apparent shortage of physicians, the country begins to expand the medical education program rapidly. This has been made through increasing the enrolment limits and by opening many new medical schools and also by introducing new teaching approaches. For instance, in the academic year 2014/2015, there were more than 14,000 medical students in 33 medical schools/colleges [4, 5]. On the contrary, there were severe shortages and poor composition of medical instructors in most medical schools and also high turnover of physicians even in the longstanding medical schools to manage medical education programs [6, 7].

Unlike that of low- and mid-level health workforce training, such a strategy and recommendation need to take into account the requirements for medical education and the local context in which the recommended strategy is being implemented, for instance, medical education workforce distribution and composition, the teaching hospitals’ capacity, patient flow, and other inputs including issues related to motivation and experience in implementing and organizing such medical education programs in the country [8–10]. Moreover, there are potential consequences, for instance, if the intended strategy is not implemented properly.

Human resources for health (HRH) development should be an integral part of and a sub-system of a country’s healthcare system. The system should put a monitoring mechanism in place to evaluate the status of human resource in time (in terms of its type and mix). The planning should be done far in advance than making it suddenly like the case of flooding strategy in Ethiopia [11]. Nevertheless, many low-income countries including Ethiopia do not have such well-organized monitoring system to detect when the crisis or the surplus can potentially occur as observed for low- and mid-level health workforce [12].

Policy makers used various techniques to overcome the apparent shortage of HRH. However, their success depends on several interrelated factors such as the capacity of the medical schools and the available medical education workforce [2, 6, 13]; the cooperation made between different stakeholders in addressing the problem; the use of evidences to project future workforce requirements (the HRH supply and requirements are two sides of the same coin); and finally the translation of lessons learnt from previous programs into practice are also essential, not to move from one side of the crisis to another; from severe shortage in quantity of medical doctors to surplus in number and/or shortage in the required skills.

In this regard, however, the rapid transition from severe shortage to massive supply to ensure the current and future physician workforce demand has become a contradicting phenomenal context which lead us to raise various questions around the issue and the context in which it has been taken place. Particularly, the capacity of medical schools to admit a massive number of medical students, the health care service demand to absorb the forthcoming medical graduates, and the quality of medical education have to be explored [2, 6].

Therefore, the purpose of this grounded theory research was to discover views and opinions of different stakeholders (government officials, academicians, individuals working with NGOs, professional associations, consultants, and researchers in the area) on how the rapid expansion on physician workforce production has been taking place, and the forces and factors that lead to its occurrence and subsequent consequences at present and in the future. Particularly, this study uncovered the views of different stakeholders on the health system response for physician workforce shortage using the so-called flooding strategy and its influence on current and future medical instructors, patients, health care system, and medical graduates.

## Methods

### Setting

The study setting is Ethiopia, East Africa. Ethiopia is a low-income country with a total population of over 93 million [14]. To capture a wider range of perspective, respondents of the particular study were selected from six regional health bureaus (Amhara, Oromia, SNNPR, Tigray, Harari, and Somali Region of Ethiopia) and two city administrations (Addis Ababa and Dire Dawa) health bureaus [15], seven medical schools (Addis Ababa, Bahir Dar, Jimma, Haromaya, Hawassa, Mekelle, and University of Gondar) with different levels of experience in medical teaching [6], and the private sector (professional associations, NGOs, researchers and consultants).

### Study design

The study adopted a grounded theory research approach, which examines the causes, contexts, consequences, and conditions of phenomenal processes (massive medical student admission) and to understand the patterns and relationships among these elements [16, 17].

### Sampling procedures

Purposive sampling strategy was employed at different phases of the data collection process [18, 19]. Initially, five individuals were selected from three different settings (academic, clinical care setting and administrative, government health bureaus). This initial phase informed us on subsequent sampling procedure to be considered, which is referred to as theoretical sampling in the grounded theory research. So that, in the second phase, the study involved government officials, medical instructors, and hospital Chief Executive Officers (CEOs). Information related to physician shortage, migration, retention, and massive production and its consequences were reached to the level of information saturation as we collected the data from six regional and two city administration health bureau officials, hospital CEOs, and academicians from seven medical schools.

Nevertheless, issues related to the role of system on HRH development such as HRH policy, strategy, planning, and capacity were emerged as new points of discussion in addition to the central phenomena. As a result, senior researchers, consultants, knowledgeable individuals who had been working in the health care system, NGOs, and professional associations were involved till information saturation was achieved. The two investigators (TA and DH) established contact with the potential study participants. None of the invitees refused to participate except one senior government officials in a study region.

### Data collection

Semi-structured interviews were used to collect the data. The main points of discussion were as follows: reasons for physician shortage, physician migration and retention, the reasons for flooding, readiness of the medical schools to admit massive number of medical students, consequences of flooding at the present and in the future, future physician workforce and their competency, job opportunity, opinions on the flooding approach, and questions related to HRH policy/strategy/planning and the like. The participants’ demographic characteristics were also collected at the end of the discussion. The interviews were conducted in Amharic language and were flexible to each of the respondent’s background and experience, and tape recorded with the permission of the respondents. Notes were taken during the discussions to follow on new emerging ideas and to refine subsequent interviews as well as the selection of the next informants. One of the investigators (TA) conducted the interviews; however, with the interest of time, the transcriptions and translations were done by other experienced individuals.

### Data analysis

Data transcription began simultaneously with the data collection. Verbatim transcription was made to all tape-recorded data and then translated into English language by the same person. Consistency of the transcribed and the translated data was checked and compared by TA. The textual data were read, edited, and organized in MS word files before being assigned to ATLAS.ti 7.0 scientific software. Each transcript was read line by line before breaking it into different categories.

The data coding process follows the procedure recommended by Strauss and Corbin [17] for grounded theory research including the use of constant comparative analysis and memoing. During open coding, code names were drawn from the words of participants; it enables to group similar events, patterns, and opinions under a common heading or classification [16]. And then similar categories were related to subcategories along the lines of their properties and dimensions, this coding process is commonly called axial coding which is very essential for clustering, categorization, and data reduction [20]. For selective coding, the purpose of this phase is to establish the central phenomena or core category of the study through integrating and refining categories. In this study, the central category is massive physician workforce production (flooding) and its consequences.

The coding procedure was made by TA and WM who evaluated naming, dimensions, and properties of the codes and finally the research team agreed on the categories and subcategories and on their relationships to be illustrated on the diagram [21, 22]. In addition, the study also benefited from the presentation made at the School of Public Health, Addis Ababa University (as external audit), and the feedback given on the preliminary findings by the study participants who were willing to do so (member check).

The findings are presented in narration with short introductory descriptions, whereas the researchers’ interpretations will be presented in the “Discussion” section. The quotations are presented in italics with a source for each quotation (respondent setting at the time of the study) then followed by the number of respondents. There are three abbreviations in the document; Ac stands for the academic setting; Gov: for government health sector; and Pr: for the private sector. Moreover, in certain circumstances, the symbol [*] is used within the body of quotations to indicate the researcher’s clarification of the meanings of some ambiguous concepts or the multi-interpretation of words.

## Results

### Demographics

This study involved 43 purposively selected individuals with varied representation of age, academic levels, years of experience within the country’s health care delivery system, and positions both in academia and public and private sectors including in local and international NGOs (Table 1).

**Table 1:** Characteristics of the study participants

Characteristics	Variable	NO
Gender	Male	39
	Female	4
Date of birth	After 1980	12
	1981–1970	16
	1969–1950	8
	Before 1950	7
Educational level	BSc	3
	GPs	5
	MD + SP/SS*	25
	MPH	4
	PhD	1
	MD + MPH	5
Affiliation	Academics (full professors)	21(4)
	Public health (senior position)	16 (5)
	Private (researchers in HRH)	6 (2)
Service years	≤ 10	17
	11 to 20	10
	> 20	16

*MD* medical doctor, *SP* specialist, *SS* sub-specialist

### Categories of the study findings

The central phenomenon of this study is massive physician workforce production within Ethiopian context. The main categories are (1) reasons for medical education expansion; (2) preparation for medical education expansion; (3) the consequences of rapid medical education expansion; (4) massive production as HRH development strategy; (5) cooperation on HRH development; (6) HRH strategies and planning; (7) system capacity for HRH development; and (8) institutional continuity for HRH development.

### Reasons for massive physician workforce production

The analysis identified two main reasons which entail the government for rapid expansion in medical education: the first one is high physician workforce demand in the country which is aggravated by high physician migration, low production of medical doctors, and expansion in health care facilities. None of the study participants denied the high demand but they have concerns regarding the way the government went about increasing the numbers using “the flooding strategy”.

*In our region, there was extreme shortage of physicians when it is compared with the figure two years ago. Their number has not been above 100 but now, there are many medical doctors who are coming to our region because of the government strategy that has started to produce a huge number of medical doctors....* (Gov3)

The second cited reason was gaining political acceptance both internally and externally:

*High migration creates negative image for the country, so the government has to give coverage by flooding large number of medical doctors, to overcome the shortages of physicians in the country.* (Ac6)

*The government should address the health demand of the community. Otherwise, it puts the existence of the government into question…, unless it produces huge number of nurses, physicians, midwives and surgeons….* (Gov4)

### Preparation on medical education expansion

The medical education expansion has to be done with due consideration of the system’s capacity. Here, two different but interlinked capacities were identified, the capacity of medical schools to enroll large number of students, and the health care service demand to absorb graduates and how to use them effectively:

*The major issue considered during the placement of students in Universities is the facility not the number of students; the number should take in to account the available resources such as number of classrooms, patient flow, medical teachers, library and IT facilities. If you accept 1000 students, side by side there are issues that have to be taken into consideration.* (Ac11)

### System’s capacity

The second point of argument was on the system’s capacity or preparation made to employ and deploy the upcoming large number of graduates and how to use them effectively:

*…If the production of medical doctors continued like this, our existing system is not ready to accommodate all of them, …although there are 800 primary hospitals, 200 general hospitals and 50 specialized hospitals that will be soon built as planned by the government… the system may not accommodate all….* (Ac6)

*…the health care system should rethink it again how to absorb 3000 graduates each year and use them effectively. If that is not the case, it should devise a way where to take them, including the change in the medical school curricula for not considering the international market*. (Pr3)

### The consequences of rapid medical education expansion

Respondents were increasingly concerned about enrollment expansion at the existing medical schools and the opening of many new medical schools. They were more concerned about the readiness of these new schools to run smoothly without any ill consequences at present, in the near future, and in the long term.

### Current consequences

Respondents from the academia explained that massive enrollment in the medical schools have been affecting the quality of patient care. There is high health service dissatisfaction, violation of the patient rights, and in some cases it could be the potential sources of treatment errors:

*How could you say that a mother should stand naked and give birth in front of 20 students? Even for the teachers how could you consider this as a good practice… as a teacher I myself do not agree, so that the policy violates patient rights* (Ac11); *If you go to wards on Tuesdays and Thursdays, there are a number of queues and mistreatments of patients. They may not come again due to the ill treatment they have received from our hospital.* (Ac13)

Furthermore, it was noted that the approach has also been affecting many aspects of the teaching-learning process. For instance, teacher–student relation, skill acquisition and teaching style:

*Yes, there is a flood of students... there is dissatisfaction; you may not know some of these students by name due to their huge number and some of them come only for exam.* (Ac15)

*…the rooms become too suffocated during clinical round so that there is a change from ‘bed side to tree side sessions’* [*observed during the data collection in one of the prestigious medical school]*.* (Ac5)

In addition, when it comes to the medical instructors, it caused more workload, caused job dissatisfaction, and affected the instructors' motivation especially for those who want to work in the public sectors:

*Most of the time, the huge number of patients may not create frustration but the large number of students who flooded to the university created unbalanced workload on medical instructors …those departments which have been teaching 60-100 students are now teaching about 300, so that, this caused the staff to raise quality issues, creates workload, and frustrations on the teachers.* (Ac9)

### Intermediate consequences

In the short term, the current medical education expansion will produce low-quality medical graduates who are incompetent and low professional value for medical service provision:

*It is good to have more human resource that satisfy the needs but we have to think of the quality of our graduates; they should not commit mistakes while providing service.* (Ac12)

*… for the coming generation whom we are producing through flooding strategy, however, it is difficult to teach the value of medicine… they will leave without knowing why and what to do.* (Ac14)

### Long-term consequences

It is anticipated to affect the system, graduates’ career, and the community. There will be surplus of physician workforce, unemployment, and pressure on the system in the long term:

*…flooding may seem a good strategy superficially but it is a dangerous. It may put the country into crisis even if good physicians are being produced; they may not get jobs, it will be very difficult after investing a lot... this happened in Mexico once in history. They flooded new medical doctors but their system could not absorb all.* (Ac17)

… ‘Speed, Volume and Quality’ should not go together at once. When you load the truck beyond its capacity, the truck can break down and cause damage to the public [* used to explain the effect of flooding], it affects service quality as well. The flooding is more than the country’s capacity (Pr5); as it happened to the other health care professionals, being unemployed for the physician workforce is going to be a reality in this country very soon.... (Ac3)

In spite of the overall physician workforce shortage in the country, during the study in Dire Dawa and Addis Ababa city administrations and to some extent in Tigray and Harari regions, the demand for general practitioners has been saturated. However, one of the government officials argued:

*… this is done based on the plan and policy direction, otherwise I do not believe that the government does this without any facts and research findings. The number and professional mixes are usually taken into consideration so that the human resources are capacitated based on the needs….* (Gov3)

### Flooding as HRH development strategy

In the study, the use of “*flooding*” as HRH development strategy was viewed as a “*forced*,” “*disastrous*,” *and* “*wrong*” strategy which can potentially lead the country in to another crisis. The following statements illustrate the respondents’ opinions in this regard:

*There is no doubt that we have the shortage; and doing something to come out of this problem with in a short time is correct. But the main question is, are we trying to satisfy our human resources needs or taking steps which make us to commit another severe crisis?* (Pr1)

*…the government forces public universities to take a huge number of students at once without equipping them with the needed resource. That is a disaster.* (Ac10); *I am saying that the strategy by which we are trying to increase the numbers is ‘wrong’.* (Ac17)

However, few government officials have different opinions about the strategy though they admit the fact that it affects the quality. One of the government officials stated:

*It may compromise quality, yes, it may. But, if you ask me about the system, yes! It is the right strategy. If you do not do this, you will not address the needs of the society.* (Gov4)

However, another respondent disagreed with the idea of merely increasing the number without the necessary medical skills:

*After making the number, number, number….so big, what if the number is not working? … It has a danger! As to me, to tell you frankly, the strategy that compromises the quality for the sake of increasing the number will not be palatable as a citizen.* (Ac17)

### Cooperation on HRH preparation

However, all stakeholders have been working for a common goal which is to improve the health service delivery in Ethiopia. This study discovered lack of mutual understanding and trust, co-operation between the government, academia, professional associations, and individual experts, and lastly the perception of differences among the actors:

*When they* [*government officials] *answer these questions* [*HRH related] *they do it by themselves or they may involve very few experts. They invite professional associations for the sake of formality; not for real. This is done for two reasons; first the policy makers create limited opportunity of participation for mutual understanding and problem solving using evidences, and second professional associations have also limited capacity…* (Pr1)

*…in the system, the bureaucracy should not ignore physicians, from my experience I learned that physicians contribute more when they get involved more….* (Ac2)

In addition, partiality, lack of dialog, and mutual trust were also mentioned as contributing factors for the existing low cooperation:

*Both* [*government officials and medical professionals] *have their own biases about one another which discourages dialogue to narrow the gap; they should be flexible to accommodate their differences.* (Ac13); *…they* [*professionals and the government] *do not work closely; they do not work together, because they are skeptical to each other.* (Ac1)

There are also concerns raised about the influence of political stances in professional and technical matters with potential source of dissatisfaction, limited professional involvement, and lack of cooperation with the system. The following statements illustrate such views:

*I have a problem to judge on this specific issue* [*political involvement] a*ccording to the Ethiopian law, the leadership position up to the level of a minister has to be assumed by members of the ruling political party. The positions below the minster must be filled with technocrats that may not be affiliated to any political party including the one in power. However, what is practically applied is not so... There might not be non-politician individuals who are fit for that position. The politicians may be the only ones who have the privilege to get it …* (Ac16)

*…the existing political elites lost trust on the educated group of the country. There is no mutual respect, sense of belongingness....* (Ac3)

### HRH strategy and plan

During the study, there was no written document on the HRH strategy or plan at the FMOH level or in the regional health bureaus. And we were told that the Federal Ministry of Health will finalize its revision very soon. However, respondents were concerned on the fact that the delay in drafting HRH strategy has been contributing for ineffective HRH development and unclear career path for health human resources. It is also cited as a reflection of the health sector’s lack of capacity:

*We have to make things clearer through the human resource policy/strategy, but still now we do not have that because we are working in urgency.* (Pr1)

*I think first there should be a plan how many physicians do we want to have in the coming ten years in the system? What type of physicians do we want to have? GPs, surgeons, OBS/GYN specialists, cardiac surgeons and so on? Ok, we need all, if so, we have to prioritize. At the same time, we have to look at the need. Really, it needs detailed analysis and plan.* (Ac2)

*…first let’s have a short term plan, which is developed and reached into consensus at all level through strong discussion and evidence. And let’s be committed not to change that direction while we implement it, either by internal or external pressure.* (Pr5)

### System’s capacity for HRH development

This study discovered that the health system has limited capacity to execute its activities such as proper HRH planning, leadership, coordination and valuing expertise. In addition, such capacities are also important to properly work with internal and external stakeholders, to have sustainable institutional functionality and to evaluate the effectiveness of the intended strategies:

*…the ministry that lead the country’s health system is staffed with very few prominent people and the vast majority, the so called officers are simply….* (Ac17)

*…things should go without losing their starting point… by focusing on their destination. For that, it needs experience…and when you do something for ten years, you provide a witness of ten years’ experience, and when you do it for 20 years it is so….* (Pr3)

In the system, high attrition and low retention negatively contributed for the system’s functional capacity:

*Institutional memory is affected by attrition in human resources from the system… activities which require high skills are usually done by seconded individuals* from UN system or NGOs [*advisors, consultants] *and their role is not in decision making… when they got better opportunity, they just leave. Thus, there is lack of institutional continuity and the system prefers to start anything as new….* (Pr5)

The role of external support to influence independent decisions was also mentioned as an additional factor:

*Since our country is poor, they* [*donors] *run everything; important decisions are made in Washington, because most of the health programs are funded by the United States Agency for International Development (USAID)…However, here the strength of the ministry is very important. If they are wise enough, the sector can harmonize its plan with the donors’ interest or be able to resist to some programs which are not in its priorities and are prescribed by partners.* (Pr2)

### Institutional continuity for HRH development

Experienced respondents were concerned about lack of functional continuity of the system. It contributes for lack of HRH strategic planning and also not to have interconnected transitions when changes are made. As a result, in the public health sector, things are short lived and there is no lesson learnt from the past:

*In HRH there should be clear strategic directions and reasons for that, but we do not have such practice. Because things are short lived and there is no institutional memory. No one looked what had been done in the past. It is simply said, oh this is changed, that is changed but no one thinks how we can link with the previous. Debates made to reach at the consensus has not been documented. Nobody asks questions in how this issues are handled in the system.* (Pr3)

*But, the system lacks sustainable institutional functionality, if it is so there is continuous in and outflow monitoring mechanisms of the health workers before the things worn out, ends in shortage or surplus.* (Pr5)

Another point which has been raised in relation to the system’s functionality was absence of uninterrupted professionalism in each political system:

*Practically, if we have clear central principle for community health needs, as the government changes, there should be a continuity of system with some modification but when we lose institutional continuity, we also lose our central principle and then we start afresh but we remain there, when you uncover things and comeback to the central principle, because the definitions and the principles might not be changed in each system.* (Pr2)

However, another respondent added that the issue of sustainability is even a concern within one government:

*…Institutional memory, let alone to have long term planning, the system cannot proceed on the issues that had been started by the successor of the former minister.* (Pr5)

## Discussion

This study discovers a variety of challenges with regard to the current physician workforce development strategy through massive admission of medical students and rapid scale up. There is mismatch between the number of students, patient flow, size of teaching hospitals, and medical instructors that lead to different forms of negative consequences currently, in the near future and in the long run. Furthermore, lack of cooperation between policy makers and medical schools (the implementers) and lack of HRH policy/strategy and planning could put the system’s effort barely successful.

Strategic response for HRH development in a country through participatory planning process is very essential [23]. However, implementing human resource production strategy in isolation either to gain political acceptance (regardless of its strong support for health results) or which is not closely aligned with medical schools’ capacity, the medical education workforce and the setup will end up unanticipated or in adverse consequences [24].

In our context, most medical teachers are clinical service providers [25] and teaching hospitals are expected to provide the highest level of care in the country. Nevertheless, the study explored that the quality of care, patient rights, and satisfaction have been contradicting with the primary aims of teaching hospitals which are delivering specialized care. Admitting large volume of students to the teaching hospitals has been affecting quality of patient care, their rights and privacy in the short and the long term. This is regarded totally inconsistent with the government’s health sector transformation plan which aims to bring quality of care and compassionate and respectful service [4, 25].

The ultimate aim of medical education is for better health care delivery through scientific, ethical, and social standards of medical education [23]. Such effective teaching also requires flexibility, good teacher–student interaction, energy, a variety of teaching methods and teaching styles, and commitment to address learners’ needs within a busy background of clinical care [26]. The current study explored major challenge across medical schools and is becoming a critical factor to produce the required human resources in diverse disciplines and settings [7]. Also, an area of critical concern for most respondents is the use of flooding strategy in medical doctors training wisely indicated as “the volume, speed and quality cannot go together”.

In fact, in the system, there were contradicting reports. On the one hand, massive expansion in health workforce preparation is reported as a success [4] and on the other hand, the inadequate knowledge and skills of health professionals during their regular training which consists of critical shortage of qualified teaching staffs in the teaching institution; and poor motivation and retention strategy were reported as a challenge [27]. Such reports provide strong support for the finding of this study, which indicated that the country has been moving from one form of crisis to another, from *shortage in quantity to scarcity in skills*.

Furthermore, in some places, there are signals which may indicate that the system cannot absorb and use the upcoming physician workforce effectively, particularly GPs. For instance, in Amhara region, GPs were assigned to different health care facilities temporarily till the new facilities’ construction was completed. Similarly, Dire Dawa and Addis Ababa city administration health bureaus reported that they did not have vacant positions for GPs. On the contrary, the medical students were not ready and had very low intention to work in remote and rural areas in the country [7]. Supportive evidence is also documented on HSDP EFY 2007 (2014/2015) annual report [27], as rightly asked by the respondents, “What if the system is unable to absorb? Even though they will be competent enough to work in the system, where do we take them?”, including the issue of curriculum change which does not consider the international market, although there is the opportunity and the demand [28] and high medical student intention to leave abroad [7].

Human resources cannot be stored or discarded. In the notion of this study, it reverts the direction of discussion back to the issue of proper context-based planning before implementing the intended strategy. And this study identified lack of clear strategy and planning for health human resources as one of the problems at all levels in the system. The only exception is the draft strategic plan which indicates the required number of physicians by 2015 (10,846 general practitioners and 5178 specialists) [25].

The problem related to planning might be more complicated when things are done in urgency, or in this case through rapid expansion and flooding. It may not capture entirely the HRH planning process including time for analyzing the situation [23]. Because at the time of this study, there is no clear HRH strategy for physician workforce which means there is no proper planning. However, the HRH production should be a result of HRH strategy (a product of lessons learnt from the past, and consulted and coordinated efforts of the present). In addition, human resources planning indicates the system’s capacity (including predictive capacity of the future using demographic, epidemiologic, labor market, and HR productivity in a fast changing environment along with the need/demand) through interconnected transitions. This is deep-rooted into another problem of the health system a “system of functional continuity” which the study discovered as a “basic” problem of the system. And issues related to *strategy*, *planning*, and *system’s capacity* are understood as “underlying” problems of the health system. This justification matches with previous report which stressed on the need for a system of continuity in Ethiopian context [29].

A system of functional continuity is one of the major requirements of the health system. In this study, it refers to the point at which things started, the direction it goes and the destination where and when to reach through interconnected transitions including the lessons. However, the study underlined that its absence can play a key role for not having HRH strategy, long- and short-term plan at all levels. The situation might be more complicated by additional factors (such as working in urgency, frequent reforms, political system changes, and lack of HR and economic capacity including other internal or external influences). For instance, lack of HRH strategic plan in the country to this day (the draft for HRH strategic plan dated 2009 to 2020 which is not officially communicated or indorsed but partly implemented) [25] is a good example. In addition, accelerated HRH production and a shift from one category to another also explains the situation very well.

On the other hand, sustainable functionality of the system also requires its own human resources with the required capacity for planning and prediction (human resource management capacity) [30]. In addition, understanding the principles of cooperation and partnership to work effectively with local people and international stakeholders might be essential and can contribute to sustainable functionality of the system. In the health system, there are also external and internal drives and interests. For instance, the intention to achieve the then MGDs is documented as a reason for flooding) [3]. On the contrary, there is also internal demand and severe physician workforce shortage which might be in favor of flooding [27]. However, the use of flooding strategy for physician workforce development in the Ethiopian context is not viewed positively instead was seen as “dangerous”, “forced” and “wrong” strategy. Thus, there should be a leadership workforce capacity for harmonizing and interpreting such influences to the local context. And in this study, the system’s capacity is considered as one of the underlying problems.

### Limitation

The views of individuals working at the Federal Ministry of Education were not included, in spite of the repeated attempts to incorporate their opinions.

## Conclusion

Medical education needs longer time of preparation; however, within the context of this study, rapid transition has been made from low production, high migration, and severe physician workforce shortages to massive production and creating many new medical schools. In relation to massive production, two distinct types of preparations were identified; preparations which need to be made in the medical schools and in the system, though at the time of the study both were not sufficiently addressed. As a result, there were potential consequences at the present. These were related to clinical service delivery, patient rights and privacy, medical education workforce, and quality of medical education. In the future, it was anticipated to affect the graduates, the system, and the community in the short- and long-term consequences of physician workforce flooding (the upcoming crisis)*.*

**Fig. 1:**
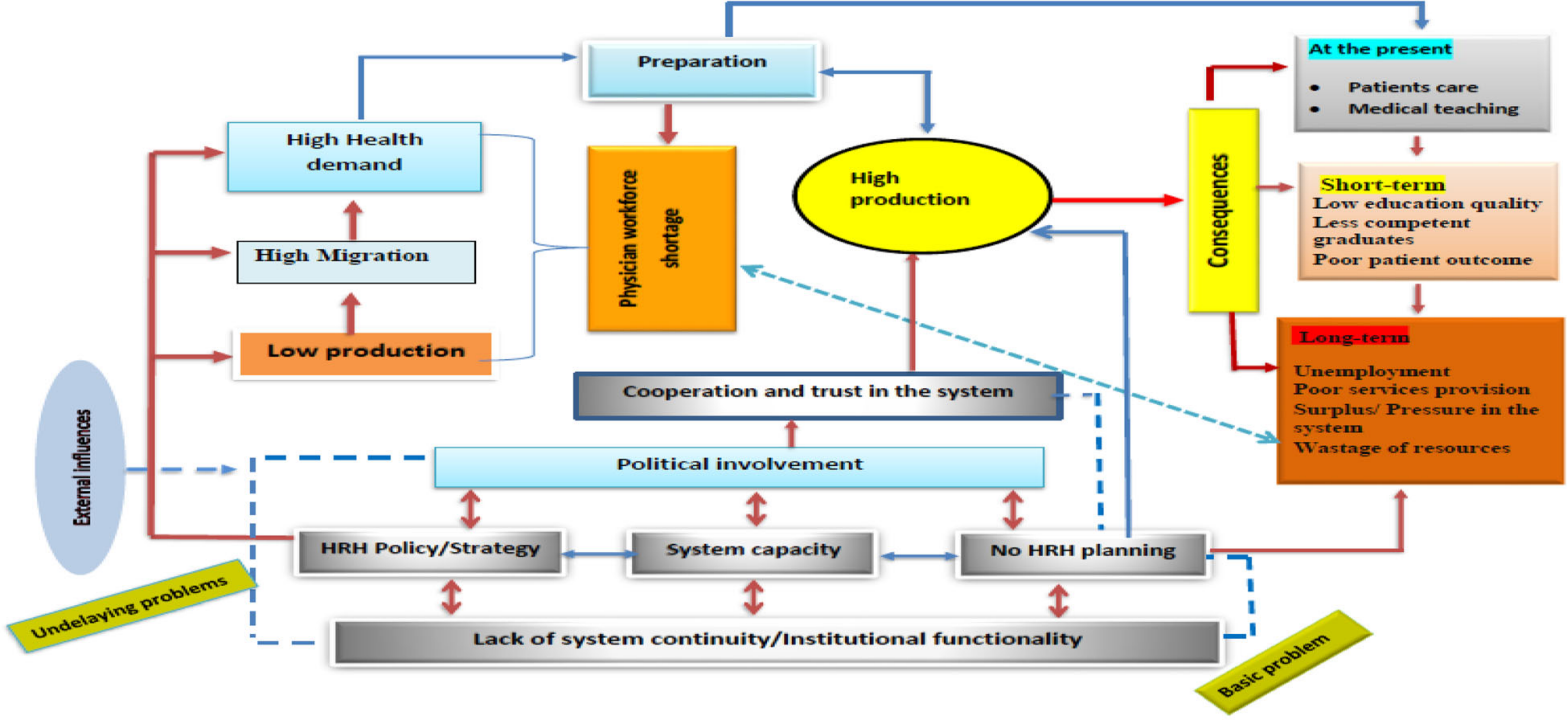
Framework for health system’s response for physician workforce shortage and its consequences in Ethiopia (constructed by the researchers)

On the other hand, massive production has been taking place in the context which lacks cooperation and mutual trust between the medical schools, professional associations, experts, and the government within highly politicized environment. Hence, though the strategy is smart enough in all aspects, the absence of cooperation and mutual trust might be a challenge for its success. Furthermore, lack of proper HRH policy or strategy and planning and human resource capacity (*the underlying problems*) to deal with the strategy and proper planning can influence one another, whereas lack of institutional functionality might be the *basic problem* behind the underlying and the apparent problems which have been taking place in the system (Fig. 1).
